# Drivers of CO_2_ Emissions: A Debt Perspective

**DOI:** 10.3390/ijerph19031847

**Published:** 2022-02-06

**Authors:** Tian Zhao, Zhixin Liu

**Affiliations:** School of Economics and Management, Beihang University, 37 Xueyuan Road, Beijing 100191, China; liuzhixin@buaa.edu.cn

**Keywords:** CO_2_ emissions, debt, decomposition, LMDI

## Abstract

CO_2_ emissions and debt accumulation are twin threats to sustainable development. To fill the gap that few studies can untangle the reasons behind CO_2_ emissions from the debt perspective, we illustrate debt can cause CO_2_ emissions through various channels. We then examined how debt-based drivers impact emission trajectories. We use the logarithmic mean Divisia index (LMDI) method to decompose the emission changes into five factors. We make decomposition analyses between different country groups to identify their respective characteristics. Further, to investigate the potential financial crisis impacts, we consider the full period 2001–2019 and two sub-periods (pre- and post-2008). The results show that the gross domestic product (GDP) is always the biggest contributor to emissions, whose effect on advanced economies saw a bigger decrease after 2008 than that on emerging economies. Debt–GDP is second only to GDP in contributing to emissions. It has a similar impact on emissions before and after 2008 for advanced economies, while it rockets after 2008 for emerging economies. Private debt financing of fossil fuels is the prominent inhibitor for both economies, especially for emerging economies. It has a stronger mitigation impact after 2008 than before for emerging economies, while has the opposite change for advanced economies. Debt structure and fossil CO_2_ intensity have relatively smaller effects on emissions. The crisis is an opportunity to promote low-carbon development. Since the COVID-19 pandemic is analogous to the 2008 crisis in terms of debt level and emission change, we provide recommendations for emission mitigation in the post-pandemic context.

## 1. Introduction

The COVID-19 pandemic has had disastrous consequences worldwide, emphasizing the significance of sustainable development [1]. Now, policymakers are deciding on recovering the economy through debt financing [2]. The pandemic has increased expenditure demands as countries strive to lessen the side effects of the crisis, while revenues have decreased due to dull growth and trade, together pushing debt burdens to a higher level [3]. However, as in former crises, only the concentration on economic recovery will exert devastating impacts on the sustainable development goals (SDGs) [4]. Debts are key to economic sustainability [5]. Meanwhile, environmental sustainability, represented by climate change due to CO_2_ emissions, should be at the heart of the economic response in the post-pandemic era [1,6,7,8]. The coordination of economy and environment is vital to the success of SDGs [9]. Therefore, the study of the interaction between debts and CO_2_ emissions is significant [10].

It has been proven that CO_2_ emissions can affect the debt system [11,12]. For instance, climate-related damages, such as floods and wildfires, will impair the fixed assets of firms, resulting in bad debt in the balance sheets of banks. Such firm insolvency damages the public budget and then will be converted into an accumulation of governmental debt. Besides, implementing CO_2_ reduction strategies will, in turn, intensify the debt burden because many green investments are financed by debts [13]. Nevertheless, there is less systematic evidence of the feedback loop from the effects of a debt burden on CO_2_ emissions. Further, the recent economic downturns have seen CO_2_ emissions plummeting. However, due to the recovery in business by the governmental endeavor, emissions will bounce back or even reach a higher level, as they have after each recession [14]. Actually, after 2008, most countries have needed to address issues pertaining to high debt levels [15,16,17]. The 2008 Great Recession shares similarities with the COVID-19 pandemic in terms of debt accumulation and CO_2_ emission changes. Therefore, by examining the nexus between debt and CO_2_ emissions that is based on the empirical analysis from the previous crisis and learning relevant experience, it will offer decision-makers policy implications on how to reduce emissions after this pandemic from the debt perspective [18,19].

Therefore, to achieve the task of carbon neutrality, this paper aims to theoretically and empirically investigate how debt-related factors impact on CO_2_ emissions. The remainder of this paper is organized as follows. Section 2 presents the literature review on the nexus between climate change and debt issues, the research gap, and the originality of this paper. Section 3 introduces different channels that how debt affects CO_2_ emissions and identifies the main factors in the decomposition model. Section 4 describes the decomposition results, and discussion from both the overall and sub-sample analyses. Section 5 presents our conclusions and policy implications.

## 2. Literature Review

With regard to the interaction between public debt and climate change, there are studies with an intertemporal perspective. Based on the premise that the debt–GDP ratio should remain fixed, Bachner and Bednar-Friedl [20] explore how climate change impacts the governmental expenditure and revenue of public budgets through a computable general equilibrium model. Similarly, Bovari et al. [13] examined how climate change could affect the overshooting of the debt–GDP threshold that is critical for the economy by integrated assessment modeling. Clootens [21] studied the nexus between the debt–GDP ratio, life expectancy, and emissions via a two-period overlapping generation (OLG) model considering the constant of private and public debts. Fodha and Seegmuller [22] use the OLG model to find the effect of green policies that are financed by public debt on capital accumulation and environmental quality. Fernández et al. [23] incorporated the carbon tax into the interaction between climate policy and public debt. Pereira and Pereira [10] and Rausch [24] suggested that the carbon taxation plays a positive role in the public budget by using carbon revenues to repay public debts.

For private debt, research focuses on the cost of debt (COD), which is the effective interest rate that companies pay on their debts [25]. Fonseka et al. [26] analyzed how the disclosure of environmental information affects the COD of Chinese energy enterprises. Palea and Drogo [12] discussed the relationship between CO_2_ emissions and the COD financing of Eurozone enterprises. Kumar and Firoz [27] explored how CO_2_ emissions affect the COD and debt financing of Indian enterprises and found that highly polluting enterprises have larger debt-financing costs. Fernández-Cuesta et al. [28] found that the firms’ efforts of CO_2_ emissions reduction can decrease their financial debt. Kempa et al. [29] empirically explored the distinctions between the COD of renewable and non-renewable energy enterprises.

The existing literature has studied how climate policy affects the debt situation. However, to the best of our knowledge, there is very little research on the feedback loop of debt to CO_2_ emissions. Kahn and McDonald [30] and Shandra et al. [31] concluded that high debt levels induce deforestation and water pollution and impede the use of renewable energy. Aubourg et al. [32] discussed how moderate debt relates to modernization and energy efficiency. Jalles [33] and Pacca et al. [34] explored the effects of systemic banking crises, sovereign debt crises, and currency crises on CO_2_, SO_2_, NO_x_, and PM_2.5_ emissions by using a panel data econometrics approach. However, they only examined the impact of the occurrence of the financial crises on atmospheric emissions, and did not focus on the nexus between debt and CO_2_ emissions. Therefore, we formulated the following hypothesis:

**Hypothesis** **1** **(H1)** **.** *The* *debt* *burden has a positive impact on CO_2_ emissions.*

With regard to the energy literature, decomposition techniques have been a particularly suitable tool to disaggregate the determinants of various indicators of CO_2_ emissions [35,36,37,38,39,40]. The emission decomposition literature generally uses conventional drivers such as the energy/carbon intensity, economic growth, demographic pressure, and the structure of the economy to decouple the change in emissions. Recently, an increased number of scholars have investigated the decomposition of CO_2_ emissions from financial perspectives. For example, Zhao et al. [41] found that investment is a positive contributor to the CO_2_ emissions in China, and the enhancement of capital productivity and investment that is directed to green activities would mitigate emissions. Shao et al. [37] used the decomposition model to analyze the effect of investment and research and development (R&D) on emissions. Yagi and Managi [42] decomposed corporate CO_2_ emissions into a cost–sales ratio, total asset–equity ratio, and the total equity to integrate corporate environmental and financial performances. However, there is no decomposition study on the driving factors of CO_2_ emissions considering debt determinants. Therefore, we formulated the following hypothesis:

**Hypothesis** **2** **(H2)** **.** *Decomposition method is suitable to analyze CO_2_* *emissions from a debt perspective.*

To fill the knowledge gap, the contribution of this paper is threefold: (i) This is the first study to compile different impact paths from public and private debts to CO_2_ emissions, and then study how CO_2_ emissions react to debt factors; (ii) to investigate the debt and CO_2_ emissions nexus, five important variables are considered from the perspectives of debt, economy, and technology; and (iii) the logarithmic mean Divisia index (LMDI) model is utilized to conduct decomposition analysis on the impact of debt-based drivers on CO_2_ emissions to investigate their contributions to emission reduction. No other study has yet used this method and we provide a new perspective to the analysis.

## 3. Methodology

### 3.1. Theoretical Framework

Debt causes CO_2_ emissions through various channels as follows:

1. Investment and consumption through public and private debt.

Public debt affects emissions in many ways [3,43]. First, variation in debt can change macroeconomic policy and GDP growth, thus greatly impacting energy use and subsequently, CO_2_ emissions [17]. Second, low-carbon projects can be constricted because of long-term fiscal policy of lessening public debt levels and public budget deficits [5,21,44,45]. Third, governments use public debt to finance large-scale infrastructure projects, most of which are high-emission projects that consume fossil fuels [46]. Because of the long-lived nature of infrastructure, it can cause a carbon lock-in effect by locking the society into a high-emission development way [47,48]. Fourth, for countries that rely heavily on energy imports, the energy demand in the domestic market generates large amounts of debts.

Private debts consist of household debt and corporate debt. The permanent income hypothesis indicates that consumers can smooth their consumption throughout their lives by access to credit. Household debt is an apparent factor to stimulate consumption, increase GDP, and hence, results in emissions. However, superfluous household leverage can also refrain consumption [49]. Corporate debt is related to CO_2_ emissions through investment [46]. The company depends on loans as a source of funds to maintain daily operations and future investment projects. Credit to the company is still concentrated on high-carbon industries, while some credit is also used for cleaning projects through green bonds.

2. Financial market development and carbon lock-in effect.

Banks are more willing to provide loans to companies that have proven their solvency. However, companies that meet these conditions mainly consume fossil fuels and have occupied a dominant position in the market network. When companies seek funding for low-carbon and renewable energy technologies, the banks will be stricter. Therefore, this feature of debt financing further strengthens the carbon lock-in effect [50]. The progress in credit market can promote the deployment of renewable energy. A developed credit market makes it easier for companies to obtain debt financing of low-carbon projects [51]. Because an advanced credit market makes banks more efficient in collecting information on companies, in supervising enterprises, and reducing the cost of information acquisition and moral hazard. Consequently, a developed credit market is beneficial to firms regarding low-carbon investments which rely on external financing [52].

### 3.2. Model

For the decomposition analysis, the LMDI model is employed. Ang [53,54] proposed two types of LMDI decomposition formula: multiplicative and additive decomposition and we chose to adopt the latter. As a climate change measure, understanding the respective contributions of each factor to the amount of CO_2_ emissions is more important than the relative changes. Therefore, we applied additive decomposition in this study. The equations that were used for the decomposition analysis are shown in Equations (1) to (7). Equation (1) represents the breakdown into the five factors of the total annual CO_2_ emissions. Equation (2) shows the difference in emissions between two periods. Equations (3) to (7) show the calculation of the impact of each factor on the total change in CO_2_ emissions. These equations are applied to eachdriving factor and time period.

Based on the literature [42,46,55], when CO_2_ emissions change is decomposed, we begin with the index decomposition analysis (IDA) identity shown in Equation (1) [56]:

(1) $$C=GDP\times \frac{D_{tot}}{GDP}\times \frac{D_{pri}}{D_{tot}}\times \frac{FF}{D_{pri}}\times \frac{C}{FF}=EG\times TL\times DS\times FP\times FI$$

where we decompose CO_2_ emissions (*C*) into five drivers: (1) EG means GDP, reflecting the economic growth; (2) TL means the total debt–GDP ratio (including public debt and private debt), reflecting the overall debt level; (3) DS means the ratio of private debt to total debt, reflecting the debt structure; (4) FP means fossil fuels consumption per private debt, reflecting the investment enthusiasm of private debt financing on fossil energy; and (5) FI means the proportion of CO_2_ over fossil energy, reflecting the fossil CO_2_ intensity and the carbon content of the fossil fuel mix.

Next, we can calculate how much contribution each factor makes to the changes in CO_2_ emissions. The total CO_2_ change (Δ*C*) between the reference year (*o*) and the target year (*t*) is expressed as a difference. The specific technique of additive decomposition is shown in Equations (2) to (7).

(2) $$\Delta C=C^{t}-C^{o}=\Delta C_{EG}+\Delta C_{TL}+\Delta C_{DS}+\Delta C_{FP}+\Delta C_{FI}$$

(3) $$\Delta C_{EG}=\frac{C^{t}-C^{o}}{ln\left(C^{t}\right)-ln\left(C^{o}\right)}\cdot ln\frac{EG^{t}}{EG^{0}}$$

(4) $$\Delta C_{TL}=\frac{C^{t}-C^{o}}{ln\left(C^{t}\right)-ln\left(C^{o}\right)}\cdot ln\frac{TL^{t}}{TL^{0}}$$

(5) $$\Delta C_{DS}=\frac{C^{t}-C^{o}}{ln\left(C^{t}\right)-ln\left(C^{o}\right)}\cdot ln\frac{DS^{t}}{DS^{0}}$$

(6) $$\Delta C_{FP}=\frac{C^{t}-C^{o}}{ln\left(C^{t}\right)-ln\left(C^{o}\right)}\cdot ln\frac{FP^{t}}{FP^{0}}$$

(7) $$\Delta C_{FI}=\frac{C^{t}-C^{o}}{ln\left(C^{t}\right)-ln\left(C^{o}\right)}\cdot ln\frac{FI^{t}}{FI^{0}}$$

### 3.3. Data

We use a dataset of 50 economies from 2001 to 2019 due to debt data availability (The 50 economies dataset that was used emitted about 85.8% of global CO_2_ emissions from fuel combustion in 2019.). The debt-related data came from total credit statistics of the Bank for International Settlements (BIS) (https://www.bis.org/statistics/totcredit.htm?m=6%7C380%7C669, accessed on 2 June 2021). The 50 economies are classified into two groups based on BIS statistics, including 29 advanced economies and 21 emerging economies (https://www.bis.org/statistics/totcredit/credgov_doc.pdf, accessed on 24 February 2021). The data for fossil energy consumption and CO_2_ emissions from fossil fuel combustion are from the Enerdata database (https://www.enerdata.net/research/energy-market-data-CO2-emissions-database.html, accessed on 11 December 2021). We set the period as the whole study period 2001–2019 due to data availability, and two sub-periods: 2001–2007 (pre-2008), and 2008–2019 (post-2008). The reason is that after the 2008 financial crisis, CO_2_ emissions saw a transient reduction and underwent a rapid retaliatory growth thereafter. Likewise, the global debt accumulated sharply following 2008. The COVID-19 recession has been the severest economic crisis since the Great Depression. The pandemic has many similar characteristics to the 2008 crisis in: debt bubble, economic slowdown, acute reduction in emissions, and potential retaliatory rebound of emissions. Therefore, the post-pandemic emissions are likely to repeat the emission trajectory of post-2008, i.e., a retaliatory rebound [18]. The next three years’ action will determine the course of emissions in the next 30 years (https://reglobal.co/the-next-three-years-will-determine-the-course-of-the-next-30-years-ieas-fatih-birol/, accessed on 16 September 2020). Therefore, through a comparative analysis of the period before and after 2008, we can fully explore the effect of debt on emissions during the crisis and provide emission reduction policy implications in the post-pandemic context.

## 4. Results and Discussion

When presenting the results, we conducted an overall analysis and a sub-sample analysis. The detailed numerical results are listed in Appendix A.

### 4.1. Analysis of CO_2_ Emission Changes

**Figure 1:**
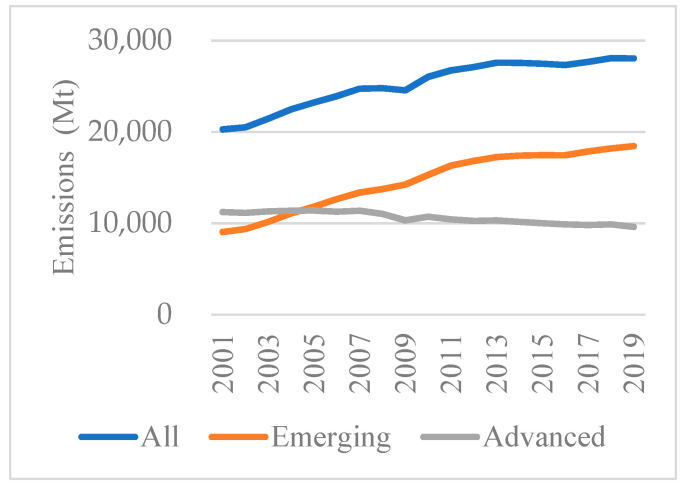
Changes in the overall CO_2_ emissions.

**Figure 2:**
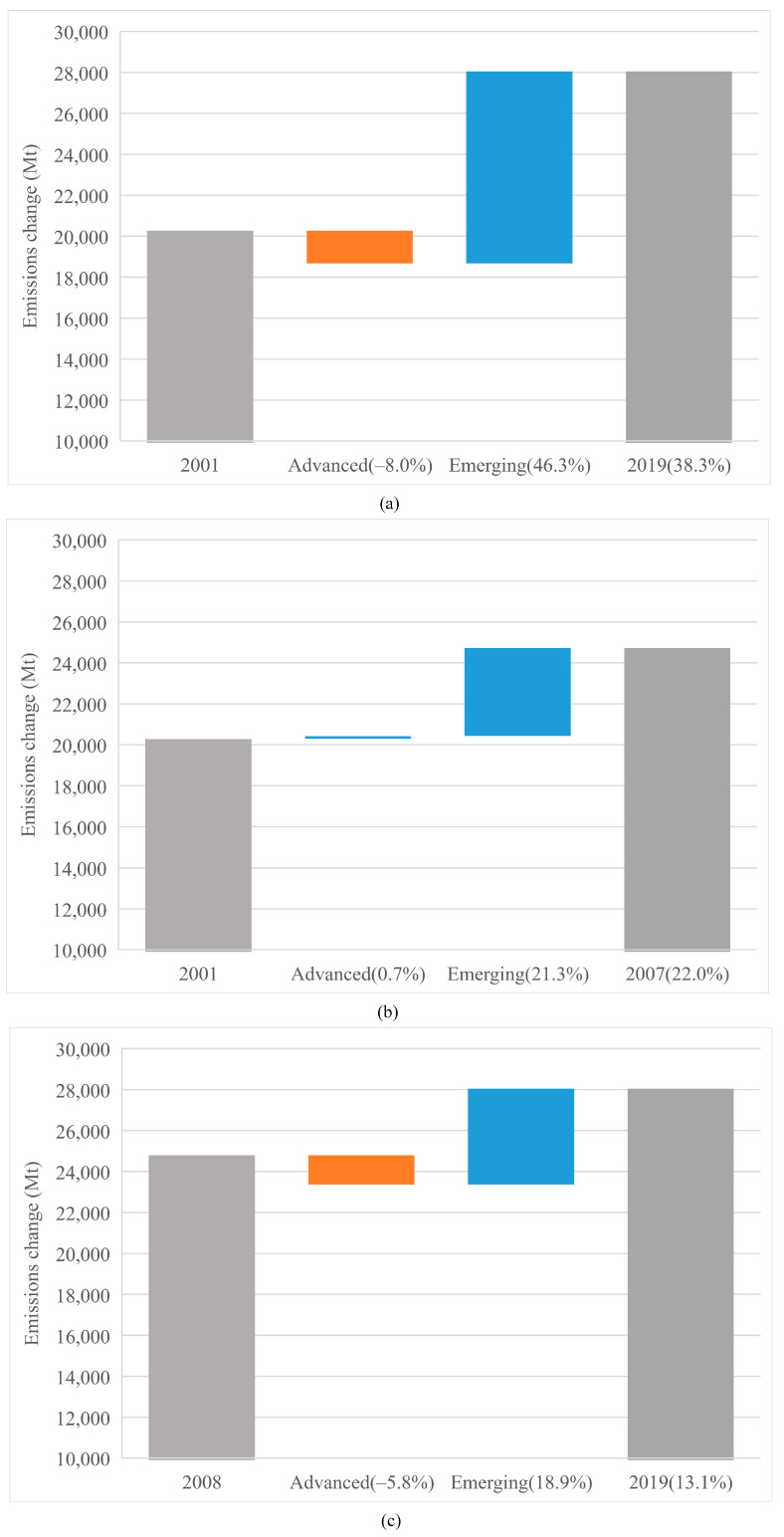
Changes in the CO_2_ emissions in various samples. (**a**) Changes in the CO_2_ emissions in various samples from 2001 to 2019. (**b**) Changes in the CO_2_ emissions in various samples from 2001 to 2007. (**c**) Changes in the CO_2_ emissions in various samples from 2008 to 2019.

The overall emission changes are shown in Figure 1. The contributions of two country groups are shown in Figure 2: the left gray bar illustrates CO_2_ emissions in a benchmark year and the right gray bar means emissions in the final year; the change from the benchmark year to the final year is represented by the bars in the middle; the values in brackets represent the growth rate of CO_2_ emissions compared to the benchmark year. The Figure 1 and Figure 2a indicate that the CO_2_ emissions accumulation from 2001 to 2019 is mainly from emerging economies (46.3%), while the advanced economies experienced a gentle reduction (−8.0%) in their CO_2_ emissions.

To analyze whether the 2008 crisis had an impact on CO_2_ emissions from the debt perspective, the overall period is classified into pre-crisis (2001–2007) and post-crisis (2008–2019). As illustrated in Figure 2b,c, the advanced economies contributed a small amount of emission increase (0.7%) during the pre-crisis period, and then make a negative contribution to emissions after 2008 (−5.8%). By contrast, we see that emerging economies maintained the dominant part in emissions in the two periods (21.3% and 18.9%), respectively, although the growth rate in the latter period exhibited a little drop.

Although crises can obviously mitigate CO_2_ emissions, this effect is very short-lived [57]. Economic activities, including large government investment and growing consumptions, were main drivers for CO_2_ emissions after 2008 in emerging countries. On the contrary, advanced economies preferred a low-carbon lifestyle after the crisis [58]. From the production-based aspect, advanced economies’ reliance on imports was reduced, thus mitigating CO_2_ emissions that are implied in international trade [59]. Thus, although the historical stock of CO_2_ emissions is mainly attributed to advanced economies, it is the emerging economies that have been responsible for the most emissions currently. Therefore, a balance should be coordinated between advanced and emerging economies [60].

### 4.2. Decomposition Results of Overall CO_2_ Emission Changes

**Figure 3:**
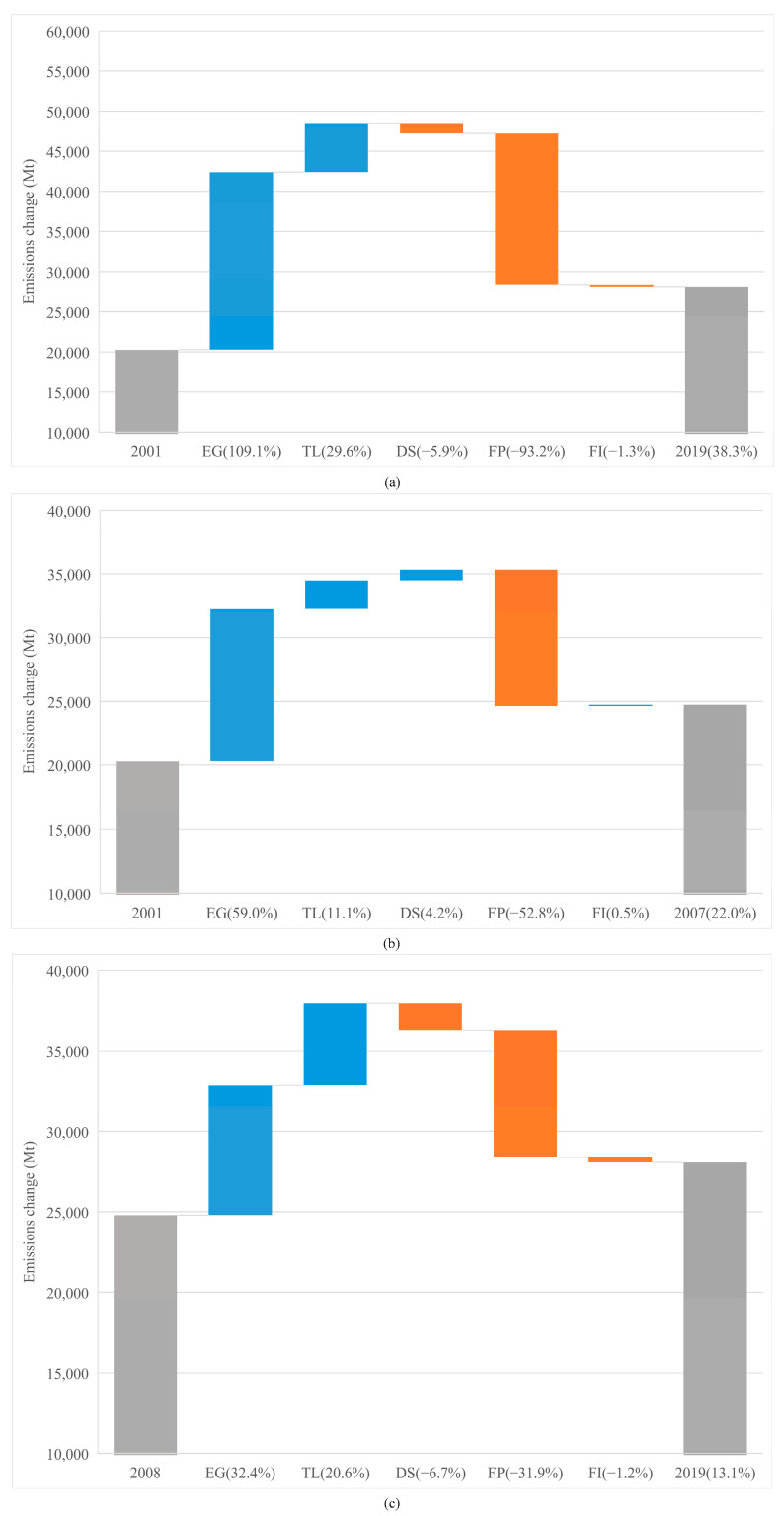
Various drivers to emission changes for all economies over the different periods. (**a**) Drivers of emission changes from 2001 to 2019. (**b**) Drivers of emission changes from 2001 to 2007. (**c**) Drivers of emission changes from 2008 to 2019.

It is necessary to explore what drives overall CO_2_ emissions, especially after the 2008 financial crisis. Figure 3 depicts impacts of the various drivers on overall CO_2_ emissions during different periods. From Figure 3a, we see that *EG* was the primary driver (109.1%) of emissions. TL was another contributor to emissions, although its effect is much smaller (29.6%) than the GDP, and the research hypothesis H1 is supported. This is consistent with [33], which states that “when hit by a debt crisis, a country experiences a rise in emissions stemming from either energy related activities or industrial processes”. However, this is inconsistent with [61], which argues that “stock markets and debt decrease CO_2_ emissions, in high-regimes of economic growth and debt.” Without lessening the FP, the overall CO_2_ emissions would have been 93% higher than that were observed. This means that the share of fossils in primary energy consumption via private debt financing has seen a significant low-carbon transformation. Further, the change in *DS* indicates that the deleverage of the private sector in the past two decades has played a helpful role in emission mitigation (−5.9%). Despite the development of low-carbon technologies, changes in FI do not make substantial contributions to lower CO_2_ emissions (−1.3%); the low-carbon mode after 2008 cannot completely counteract the outcome of the high-carbon development before 2008.

Figure 3b,c show the different parts that the factors play in the two sub-periods. First, the stimulus impact of EG on CO_2_ accumulation is more pronounced before the crisis (59.0%) than after the crisis (32.4%). The 2008 crisis brought great harm to the world economy. Although the economy recovered later, it was unable to return to the state of rapid growth before the crisis in the short run, which also suppressed emissions to a certain extent [62]. Nevertheless, the TL plays a stronger role in raising CO_2_ emissions in the 2008–2019 period as compared to the 2001–2007 period. Global indebtedness, as a major outcome of stimulus to EG, has soared since 2008, which can produce more emissions by multiple channels, and the same is true for the FP. However, the difference in the FP between the two sub-periods is higher than that of the TL. Due to side effects of the 2008 crisis, low-carbon infrastructure investments were delayed dramatically. Therefore, the credit markets are needed to improve for green debt financing. The DS that caused overall CO_2_ emissions before the crisis, played the opposite role after the crisis. This is mainly attributed to the deleveraging of the private sector. It is thus significant to monitor dynamics in private sector indebtedness, the risk of debt overhang and any consequence that is related to high deleveraging needs. The influence of FI after 2008, when switching to low-carbon technologies, was beneficial to decreasing the overall CO_2_ emissions by 1.2% during the 2008–2019 period compared with not switching.

### 4.3. Decomposition Results in Different Country Groups

**Figure 4:**
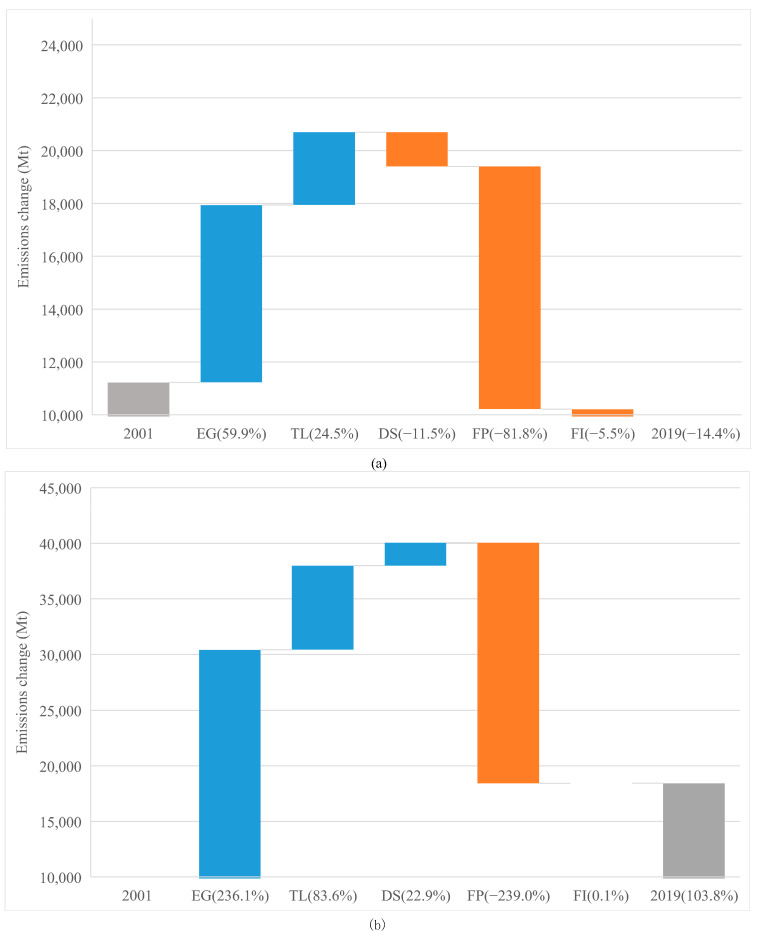
Drivers of emission changes in the different economies from 2001 to 2019. (**a**) Drivers of emission changes in the advanced economies. (**b**) Drivers of emission changes in the emerging economies.

**Figure 5:**
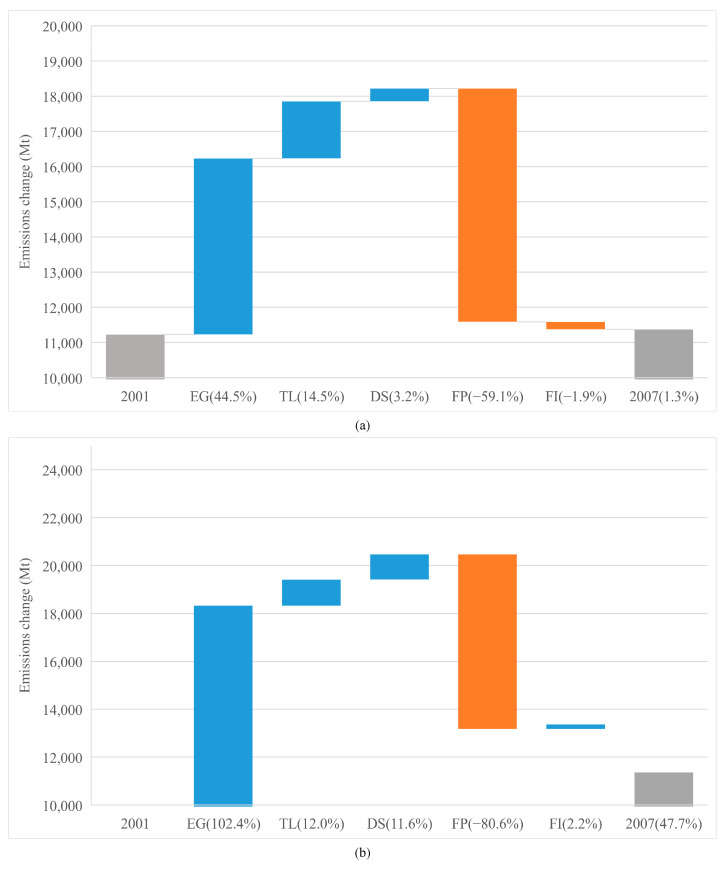
Drivers of emission changes in the different economies from 2001 to 2007. (**a**) Drivers of emission changes in the advanced economies. (**b**) Drivers of emission changes in the emerging economies.

**Figure 6:**
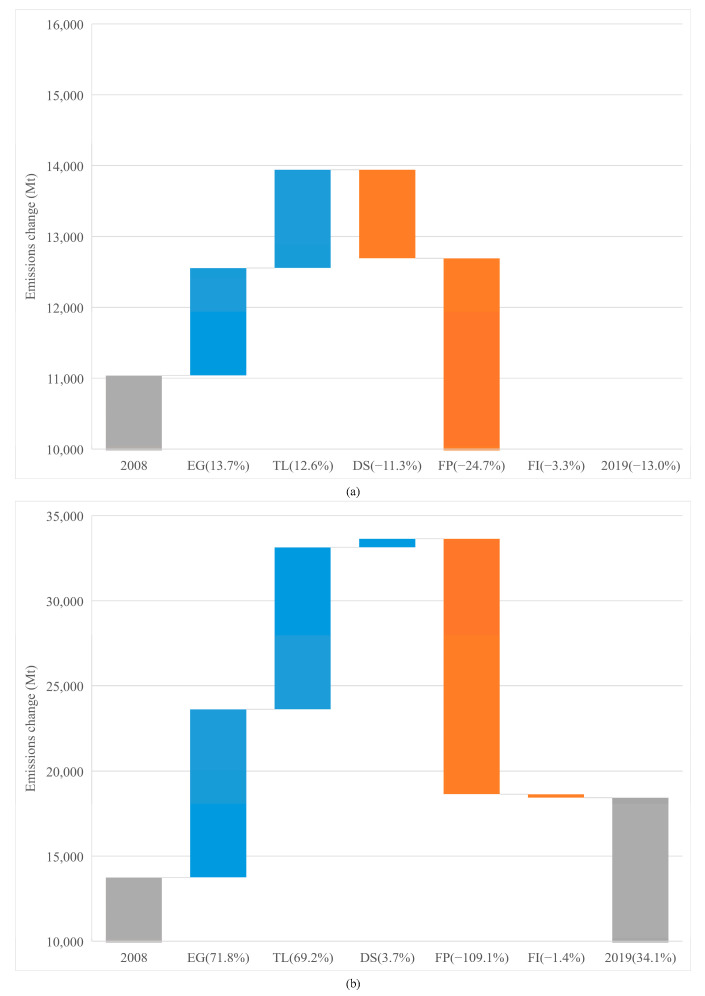
Drivers of emission changes in the different economies from 2008 to 2019. (**a**) Drivers of emission changes in the advanced economies. (**b**) Drivers of emission changes in the emerging economies.

In this subsection, we examine the impacts of the various drivers on changes in CO_2_ emissions during the different periods. The decomposition results of CO_2_ emissions from advanced and emerging economies over periods of 2001–2019, 2001–2007, and 2008–2019 are shown in Figure 4, Figure 5 and Figure 6. The research hypothesis H2 is supported because we can indeed analyze the CO_2_ emissions from a debt perspective by means of an LMDI decomposition model. In general, from 2001 to 2019, the contributor to emissions for advanced economies were EG (59.9%) and TL (24.5%), and the impediment to emissions for advanced economies were FP (−81.8%), DS (−11.5%), and FI (−5.5%). For emerging economies over the period 2001–2019, the CO_2_ emissions were mainly driven by EG (236.1%), TL (83.6%), DS (22.9%), and FI (0.1%), and the CO_2_ emissions were mainly curbed by FP (−239.0%). The discussion of each driver is as follows.

#### 4.3.1. Economic Growth

As shown in Figure 4, Figure 5 and Figure 6, for both economies, the EG contributed the highest portion of emission increase in each period, although its effects weakened after 2008. However, the effect of EG on emissions in advanced economies saw a bigger decrease (from 44.5% to 13.7%), than that in emerging economies (from 102.4% to 71.8%). This reflects that after 2008, economic stimuli on advanced economies changed the trajectory of emissions through investments towards green infrastructure. In addition, after the crisis, advanced economies were inclined to have a low-carbon lifestyle, while emerging economies were still locked into high-carbon activities to meet the basic life requirements of the citizens. Meanwhile, the import need from emerging economies increased faster than that from the advanced ones [62].

#### 4.3.2. Total Debt-to-GDP Ratio

From Figure 4, Figure 5 and Figure 6 we can see that the prominent change is the impact of TL on CO_2_ emissions. We see that whether in advanced or emerging economies, the positive impact of TL on emissions is second only to GDP. The difference is that the TL of advanced economies has basically the same impact on emissions before (14.5%) and after (12.6%) 2008. However, the impact of emerging economies’ TL on emissions has rocketed from 12% (pre-2008) to 69.2% (post-2008).

After 2008, the global debt was the highest in history. Debt expansion is remarkable in the non-financial private sector, followed by the public sector. Public debt is a powerful tool to address capital over-accumulation and enhance infrastructure construction [21]. Public debt policy, in most cases, tends to push debt-based economic activity to combat economic, environmental, or social crisis and extreme events with deficit spending. In many cases, the policy ignores the reduce debt balances after the crisis or extreme events [63]. In most advanced economies, many infrastructures are too old and need to be replaced through substantial investment. However, the majority of these investments is not sustainable, heightening the vulnerability to environmental catastrophe and debt burden. Although debt financing is necessary for infrastructure, measures are taken to counteract CO_2_ emissions by transferring the traditional high-carbon investment to low-carbon investment. For advanced economies, more green growth policies are proposed after 2008. Low-carbon infrastructure is promoted by debt financing, such as issuing green bonds and subsidies to stimulate firms to develop more green-related job positions [17,64,65].

We found that the contribution of TL in emerging economies to CO_2_ emissions is significantly higher than that in advanced economies. Developing countries borrow heavily to promote economic development and large-infrastructure investment [9]. Developing countries also face a much larger effect on the changes in budget balances following an extreme event than advanced economies [66]. The likelihood that government debt increases in developing countries after extreme events is also generally supported by the empirical literature [67,68,69,70]. It is difficult for emerging economies to coordinate economic development and emission control due to resource constraints. The massive investments in infrastructure are being driven by rapid urbanization, population growth, and industrialization of emerging economies. Further, governments of emerging economies are inclined to not invest in low-carbon infrastructure under the crisis [71]. These all explain why the TL in emerging economies plays a stronger role in emissions after 2008.

#### 4.3.3. Debt Structure

As shown in Figure 4, Figure 5 and Figure 6, the effects of DS on emissions are significantly different between the two economies. Over the 2001–2019 period, DS contributed to the decrease (−11.5%) in CO_2_ emissions in advanced economies but increased (22.9%) in emerging economies. Specific to the two sub-periods, advanced economies see the acceleration (3.2%) of emissions before 2008 and the impediment (−11.3%) to emissions after 2008. For emerging economies, both the sub-periods see a positive stimuli of DS to emissions, while the post-crisis period sees a smaller contribution (3.7%) than the pre-crisis period (11.6%). This is consistent with [46]. A total of two-thirds of debt are private sector liabilities, the excessive level of which will pose huge risks. Advanced economies have made progress in the deleveraging of the private sector after 2008. However, loose financial conditions have led to a sharp increase in the private sector leverage in emerging economies. The net capital flowing into emerging economies is an important source of external financing. However, the volatility and procyclicality of these capital flows make macroeconomic management complicated and accumulate debt accordingly, exacerbating financial vulnerabilities as a result. The instability of the financial market cannot promote greater financing for the low-carbon industry at lower costs (https://www.imf.org/en/Publications/FM/Issues/2020/09/30/october-2020-fiscal-monitor, accessed on 30 September 2020).

#### 4.3.4. Private Debt Financing of Fossil Fuels

From Figure 4, Figure 5 and Figure 6, we can see that for both economies at different periods, the FP makes the largest contribution to emission reduction. The difference is that FP has a greater effect on emission reduction in emerging economies throughout the whole period (−239.0%) and has a stronger effect after 2008 (−109.1%) than before (−80.6%). In contrast, for advanced economies, FP is responsible for the largest reduction in the whole period (−81.8%), but after 2008, its emission reduction effect is smaller (−24.7%) than before (−59.1%). Therefore, in terms of FP, the crisis affects emissions differently in advanced and emerging economies.

Firms from advanced economies face the pressure from environmental regulatory authorities. Carbon reduction has become an important business [28,72]. Private financing is becoming increasingly important for the diffusion of renewable energy. The implementation of effective carbon pricing policy is an attractive way to alleviate emissions from the private debt financing of high-carbon projects [6]. Carbon tax can also change consumer behavior, which can reduce household loans to purchase energy-intensive products. Taxation is one of the best alternative tools for this purpose with additional benefits for governments [73]. Revenue from carbon tax can help the government maintain fiscal sustainability and make public debt under dangerous threshold levels [24,74]. So far, however, carbon tax is only prevalent among advanced economies.

Relying on public investment alone is not sufficient to provide enough capital that is required for renewables development. Therefore, the financial sector is needed to provide the necessary assistance in financing. A developed financial market provides convenience for enterprises and households to invest in and consume low-carbon projects and products [75]. This is consistent with [52,76]. Advanced economies have a lower carbon-intensive lifestyle, so that FP has the most significant inhibitory effect on emissions. However, the 2008 crisis caused severe damage to the financial systems of advanced economies, including their carbon market, which also answers why the inhibitory effect of this factor after 2008 was smaller than before.

For developing countries, carbon markets are being built. This can promote low-carbon progress to inhibit the contribution of private debt financing to emissions. Besides, green bonds provide investors with opportunities in market-traded debt and listed equity securities [71]. Globally, emerging financial markets were growing the fastest after 2008. By 2015, the International Bank for Reconstruction and Development (IBRD) had issued considerable green bonds, supporting many climate-friendly projects in emerging economies. Likewise, the International Finance Corporation (IFC), the world’s largest organization focusing on the growth of private sectors in emerging economies, provides commercial loans, especially green bonds to investors.

Moreover, the financial systems of emerging economies are growing rapidly, providing convenience for investors to invest in green energy by debt financing from the domestic credit market [77,78]. The financial system of emerging economies has been able to maintain the benefits of continuous emission reductions because it was less affected by the 2008 crisis. Since the emission reduction effect of FP in emerging economies is significant, in the future, more international investors should be attracted to give credit support to the private sector in emerging economies.

#### 4.3.5. Fossil CO_2_ Intensity

FI makes a negative contribution to emissions for advanced economies (−5.5%) and has almost no impact on the emissions for emerging economies (0.1%), as shown in Figure 4. For advanced economies, from Figure 5a and Figure 6a we can see in both sub-periods, it impeded emissions and the effect is bigger in 2008–2019 (−3.3%) than that in 2001–2007 (−1.9%). From Figure 5b and Figure 6b we see that for emerging economies, after experiencing the positive effect (2.2%) in 2001–2007, FI had an opposite impact (−1.4%) in 2008–2019. The same point for both economies is that the effect of FI on the emission reduction was better in the post-2008 period than in the pre-2008 period. This is consistent with [8]. Although FI had a smaller impact on emissions compared to the other factors, it cannot be ignored.

The financial crisis is seen as a valuable opportunity to reduce emissions because it will promote low-carbon technological innovation and the implementation of green policies. Variations in FI signify the fuel quality in the total fossil fuel mix. FI can decline emissions as follows: switching towards lower-carbon fossil fuels such as from coal to gas; decrease in the carbon content of fossil fuels; and the promotion of carbon capture and storage technology (CCS). These can counteract CO_2_ emissions caused from fossil fuels by reducing FI, however, it is challenging to reduce emissions from FI. Although countries are working hard to implement low-carbon combustion technologies, coal combustion is cheaper than natural gas and thermal power plants will still use coal as the primary fuel. For CCS, although it has been proposed very early to be the most effective low-carbon technology, its commercial promotion is still in its infancy. This explains why FI is directly related to emissions, but its reduction has little effect. Further, advanced economies grasp cutting-edge low-carbon technologies, while emerging economies are relatively backward in this regard. This answers why FI has less effect on emission reductions in emerging economies than in advanced economies.

## 5. Conclusions

This paper provides new evidence for the interaction between debt and CO_2_ emissions. Based on the data of 50 economies from 2001 to 2019, this paper uses an LMDI method to examine the effects of debt drivers on CO_2_ emissions in all economies, advanced economies and emerging economies, respectively. We also studied the drivers of different aspects of economic growth and the fossil CO_2_ intensity for the comprehensiveness of our decomposition analysis. The results showed that in any period, whether for the full country sample or the sub-samples, economic growth (EG) and total debt–GDP ratio (TL) contributed to CO_2_ emissions, and the use of fossil fuel per private debt (FP) mitigated emissions. The contribution of TL in emerging economies to emissions was much greater than in advanced economies, especially after 2008. Further, the FP has the greatest effect on reducing emissions in both economies, especially for emerging economies. The debt structure (DS), i.e., the ratio of private debt to total debt, reduced emissions of advanced economies, especially after 2008, while the DS of emerging economies has always led to an increase in emissions. Lastly, the fossil CO_2_ intensity (FI), has a smaller effect on emissions.

These results can provide beneficial implications for policy-makers. First, the crisis can be regarded as an opportunity to promote low-carbon technological innovation and the implementation of green policies. As for economic development, the switch to green growth mode after a crisis should be highlighted. Governments should use public debt to finance more low-carbon infrastructure to counteract the negative impact of extreme events or crises on emissions. In particular, the debt-for-nature swap and climate debt are two effective methods for emerging economies to reduce emissions from a debt angle. As for the private level, carbon pricing and green bonds are beneficial to mitigate emissions. All economies should develop carbon tax (increasing fiscal revenue to reduce debt financing meanwhile mitigating emissions) and green bond market to counteract the contribution of debt to emissions. Advanced economies should continue to maintain the deleveraging of the private sector, while emerging economies should actively engage in deleveraging of the private sector. Emerging economies should improve their financial markets because the more developed the financial markets, the more efficient the debt financing of low-carbon projects. Emerging economies need to develop decarbonization technologies for fossil energy combustion, such as improving coal combustion efficiency and developing CCS technology. Developed economies should provide technical and financial assistance to emerging economies to jointly achieve carbon neutrality.

Although this paper demonstrates original findings, the limitation of the study still remains. This paper only conducts a cross-country study on the contributions of debt-related factors to CO_2_ emissions from a general perspective. The country-specific analysis on one economy is needed to be explored in a future study. In addition, this paper studies the effect of total debt (including public and private debt) on CO_2_ emissions in one decomposition model at the same time. Further research should pay attention to the respective contributions of public debt or private debt on CO_2_ emissions by different specific ways of decomposition. Last but not least, it is worthwhile to study the interactive nexus between debt and emissions from the perspective of extreme events or crisis.

## Data Availability

The debt-related data come from the total credit statistics of Bank for International Settlements (BIS), see https://www.bis.org/statistics/totcredit.htm?m=6%7C380%7C669, accessed on 6 December 2021. The data for fossil energy consumption and CO_2_ emissions from fossil fuel combustion are from the Enerdata database, see https://www.enerdata.net/research/energy-market-data-CO2-emissions-database.html, accessed on 1 December 2021.

## Appendix A

**Table A1:** Decomposition results of all reporting economies over 2001–2007, 2008–2019, and 2001–2019.

Period	2001–2007	2008–2019	2001–2019
Emissions change (Mt)			
Overall change	4457.5497	3258.6302	7770.3296
EG	11,956.55778	8033.500576	22,129.67539
TL	2245.826263	5100.799726	5996.376564
DS	857.0272277	−1665.344649	−1194.180518
FP	−10,707.84426	−7900.809573	−18,904.83463
FI	105.9826914	−309.5158801	−256.7072098
Contribution (%)			
Growth rate	21.98118938	13.14460529	38.31725903
EG	58.96050042	32.40539359	109.1264525
TL	11.07467908	20.57551639	29.56949394
DS	4.226195795	−6.717638009	−5.8887752
FP	−52.80281061	−31.87014695	−93.22403071
FI	0.522624708	−1.248519723	−1.265881521

***Note:*** EG, TL, DS, FP, FI refer to GDP, total leverage ratio, debt structure, fossil fuel consumption per private debt, and fossil CO_2_ intensity, respectively.

**Table A2:** Decomposition results of advanced economies over 2001–2007, 2008–2019, and 2001–2019.

Period	2001–2007	2008–2019	2001–2019
Emissions change (Mt)			
Overall change	144.3742	−1432.0529	−1620.9299
EG	5000.371759	1516.8455	6719.956381
TL	1628.094023	1386.408945	2753.796994
DS	361.2583292	−1248.306826	−1293.506313
FP	−6632.332388	−2728.045923	−9189.208458
FI	−213.0175232	−358.9545966	−611.9685034
Contribution (%)			
Growth rate	1.28590544	−12.97318793	−14.43722338
EG	44.53707966	13.74133716	59.85299635
TL	14.50103246	12.55969231	24.52739157
DS	3.21763896	−11.30860392	−11.52094214
FP	−59.07255103	−24.71378846	−81.84601643
FI	−1.897294613	−3.251825013	−5.450652731

**Table A3:** Decomposition results of emerging market economies over 2001–2007, 2008–2019, and 2001–2019.

Period	2001–2007	2008–2019	2001–2019
Emissions change (Mt)			
Overall change	4313.1755	4690.6831	9391.2595
EG	9273.185272	9869.018593	21,372.98472
TL	1088.954524	9512.143944	7567.51289
DS	1047.766564	509.9134932	2068.455571
FP	−7293.868647	−15,006.58231	−21,630.9509
FI	197.1377872	−193.8106167	13.25721402
Contribution (%)			
Growth rate	47.6515345	34.10892446	103.7537021
EG	102.4492298	71.763878	236.1266122
TL	12.03066141	69.16881664	83.60513073
DS	11.57562092	3.707903614	22.85209169
FP	−80.58193631	−109.1223542	−238.9765969
FI	2.177958692	−1.409319612	0.146464384
